# A model for promoting physical activity among rural South African adolescent girls

**DOI:** 10.3402/gha.v8.28790

**Published:** 2015-12-16

**Authors:** John Kinsman, Shane A. Norris, Kathleen Kahn, Rhian Twine, Kari Riggle, Kerstin Edin, Jennifer Mathebula, Sizzy Ngobeni, Nester Monareng, Lisa K. Micklesfield

**Affiliations:** 1 Epidemiology and Global Health Unit, Department of Public Health and Clinical Medicine, Umeå University, Umeå, Sweden; 2 MRC/Wits Developmental Pathways for Health Research Unit, Department of Paediatrics, Faculty of Health Sciences, University of the Witwatersrand, Johannesburg, South Africa; 3 MRC/Wits Rural Public Health and Health Transitions Research Unit (Agincourt), School of Public Health, Faculty of Health Sciences, University of the Witwatersrand, Johannesburg, South Africa; 4 INDEPTH Network, Accra, Ghana; 5 Department of Nursing, Umeå University, Umeå, Sweden

**Keywords:** South Africa, adolescence, girls, physical activity, transition, health intervention

## Abstract

**Background:**

In South Africa, the expanding epidemic of non-communicable diseases is partly fuelled by high levels of physical inactivity and sedentary behaviour. Women especially are at high risk, and interventions promoting physical activity are urgently needed for girls in their adolescence, as this is the time when many girls adopt unhealthy lifestyles.

**Objective:**

This qualitative study aimed to identify and describe facilitating factors and barriers that are associated with physical activity among adolescent girls in rural, north-eastern South Africa and, based on these, to develop a model for promoting leisure-time physical activity within this population.

**Design:**

The study was conducted in and around three secondary schools. Six focus group discussions were conducted with adolescent girls from the schools, and seven qualitative interviews were held with sports teachers and youth leaders. The data were subjected to thematic analysis.

**Results:**

Seven thematic areas were identified, each of which was associated with the girls’ self-reported levels of physical activity. The thematic areas are 1) poverty, 2) body image ideals, 3) gender, 4) parents and home life, 5) demographic factors, 6) perceived health effects of physical activity, and 7) human and infrastructural resources. More barriers to physical activity were reported than facilitating factors.

**Conclusions:**

Analysis of the barriers found in the different themes indicated potential remedial actions that could be taken, and these were synthesised into a model for promoting physical activity among South African adolescent girls in resource-poor environments. The model presents a series of action points, seen both from the ‘supply-side’ perspective (such as the provision of resources and training for the individuals, schools, and organisations which facilitate the activities) and from the ‘demand-side’ perspective (such as the development of empowering messages about body image for teenage girls, and encouraging more parental involvement). The development of physical activity interventions that incorporate this supply- and demand-side model would represent an additional tool for ongoing efforts aimed at tackling the expanding non-communicable disease epidemic in South Africa, and in other resource-constrained settings undergoing rapid health transitions.

Physical inactivity has been identified as a major cause for deaths related to non-communicable diseases (NCDs) worldwide (1). It increases the risk of numerous major NCDs, including coronary heart disease, type 2 diabetes, and several cancers (2), and it also acts as a key determinant of overweight and obesity, both of which also have direct causal associations with NCD risk (2–4). A recent review by Milton et al. (5) has confirmed that the health benefits of physical activity previously shown in epidemiological research from high-income countries are also to be found in low- and middle-income settings.

An estimated 31% of the world's population is deemed to be physically inactive (i.e. engaging in less than 30 min of moderate-intensity physical activity on at least 5 days a week, or 20 min of vigorous-intensity physical activity on at least 3 days a week) (6). Globally, physical activity among young people in particular seems to be decreasing over time, with a lower overall level of activity in physical education classes, lower levels among adolescent girls than adolescent boys, and decreasing levels among girls after puberty (7). With its high and increasing prevalence, physical inactivity has been recognised as a ‘global public health priority’ that urgently needs to be addressed (1).

There is, however, great variability between countries. A 2002–2003 survey that included 18 African countries showed a wide range in the prevalence of physical inactivity among African women and men, with the lowest levels in the Comoros Islands (4 and 2%, respectively) and the highest levels in Mauritania (72 and 53%, respectively). South Africans were among the least active on the continent, with 48% of women and 45% of men being physically inactive (8). Trends over time are also worrying, with the prevalence of overweight among South African female adolescents increasing from 24 to 29% between 2002 and 2008, and obesity rates in the same group rising from 5 to 7.5% (9). Further, data from the 2008 National Youth Risk Behaviour Survey found that 41% of the respondents (46% of females and 37% of males) had insufficient or no physical activity in the week prior to the survey (10). No national-level data have been published since 2008, but a recent synthesis of smaller studies conducted in different parts of the country over the past few years indicates that the situation today is the same, if not worse than it was then, with children and youth spending progressively more time in front of screens, and more than 50% of young people not meeting the recommended levels of physical activity (11). These data are of real concern for the country given that future patterns for adult health are largely established during childhood and adolescence (12).

A recent review article on the determinants of obesity in black South African women highlighted the importance of socioeconomic status and the built environment as factors influencing physical activity (13). A study in rural South Africa showed, similarly, that lower socioeconomic status was associated with less sedentary time, more walking for transport, and lower levels of moderate and vigorous physical activity (14). Puoane and Mciza (15) also reported that poor environmental conditions such as a high crime rate and overcrowding can contribute to low levels of physical activity. Cultural factors can act as additional barriers to physical activity in black South African women, such as the non-acceptability of wearing tight-fitting clothing when participating in sport, as well as the perception that taking part in leisure-time physical activity takes time away from household chores (13). Another factor contributing to low levels of physical activity in the country has been the devastating HIV epidemic, which, over recent years, has relegated other health problems in the country to somewhat lower levels of perceived importance within the policy sphere (16).

To our knowledge, the roles of culture, ethnicity, and body image, as determinants of physical activity, are not comprehensively described within the literature. There also remains a dearth of evidence on effective physical activity interventions for adolescents. This applies globally (17), to sub-Saharan Africa as a whole, and specifically to South Africa (18).

The aim of this qualitative study was to identify and describe facilitating factors and barriers that are associated with physical activity among adolescent girls in a rural area of South Africa; and, based on these, to develop a model for promoting leisure-time physical activity within this population. Our study area is in the midst of rapid social, demographic, and epidemiological transitions, with NCDs contributing substantially to the overall disease burden, alongside persistent undernutrition and infectious diseases (19). This is a situation shared by many other parts of sub-Saharan Africa (20), and as such, the model could have relevance in other settings that face similar resource constraints and that are also undergoing health and social transitions.

## Theoretical framework

In this paper, we draw on two theoretical concepts that are relevant for the effective promotion of physical activity among adolescent girls in rural South Africa: cultural competence and social identity theory.

Our understandings and knowledge are specific to, and dependent upon, the particular culture and time in which we live (21). This has important implications for the development of interventions aimed at tackling deep-rooted health-related habits within a society, since the understandings on which these habits may be founded can, and do, change over time. Recognition of the dynamic and malleable nature of these understandings makes it possible to effectively introduce new, health-promoting ideas into a society.

However, any individual or organisation seeking to engage in health-promoting activities needs to possess a high degree of cultural competence. Cultural competence is described as the ability to understand the attitudes, beliefs, and values of a given culture (22). Acquiring competence in a particular culture requires developing insights into the social, economic, and political context in which one is working. In this rural South African context, there are three major factors which frame and bring particular challenges to people's daily lives: gender inequality (23). Any health promotion intervention targeting this particular context needs to acknowledge and take these factors into account.

It is also important when working with adolescent girls to recognise the potential power of peer pressure in shaping their desires and practices. Social identity theory argues that individuals define themselves in terms of their social group memberships. Belonging to any group, whether a peer group at school, a political party, or a religion, confers some degree of social identity, or a shared representation of who one is and how one should behave (24). Just as with the major contextual factors mentioned above, the potential influence of social groups must also be acknowledged and taken into account in the development of any intervention.

## Methods

### Setting

The study was conducted within the Agincourt sub-district of rural Mpumalanga province, north-eastern South Africa, where a health and socio-demographic surveillance system (HDSS) has been operating since 1992 (25). Several study cohorts are currently nested within the population under surveillance, alongside an expanding portfolio of intervention research across the life course, with a major focus on critical problems affecting the health and well-being of children and adolescents. The Agincourt area is broadly representative of the most marginalised rural communities in South Africa. About one-third of the people living there have their roots in Mozambique (25), with an increasing proportion legally registered in South Africa.

Prior to commencing, the Agincourt community engagement team – which works to ensure good links between the community and the research projects being run out of the HDSS – discussed the study with community leaders and with the local public education authorities. All studies conducted by the Agincourt HDSS go through a ‘community entry’ process before field work can begin, and key findings from the studies are shared with the community as well as with decision makers at the sub-district, local government, and provincial level. This systematic community engagement process has, over the years, helped to build mutual trust and respect between the Agincourt scientists and the community, and, in a number of cases, it has also facilitated the implementation of interventions that have arisen out of research conducted in the area (25).

### Sampling and study population

Once permission to go ahead with the study was obtained from community leaders and the education authorities, participants were recruited through three secondary schools in the Agincourt sub-district. While the schools were selected to represent the geographical diversity of the community, they all fell within the bottom two quintiles of academic performance for schools in the country. Participants included:

1. **Fifty-one adolescent girls**, aged 13–19 years. These respondents took part in six focus group discussions (FGDs), consisting of around eight participants each, with two FGDs held in each of the three schools. The participants were stratified by age, with one FGD in each school comprising 13- to 15-year-olds, and the other comprising 16- to 19-year-olds. An announcement about the study was made in each school by the respective head teacher, and girls who wished to participate were invited to write their name on a piece of paper and deposit it into a sealed ‘post box’. There were too many applicants at one school, so a random sampling approach was used in this case.
2. **Seven adult key informants**, comprising sports teachers and youth leaders with knowledge and experience of sports and other activities of adolescent girls in the area. These respondents were recruited purposively from the same schools and the surrounding areas to take part in qualitative interviews. They included six men and one woman, ranging in age from 21 to 44 years. All of those whom we approached agreed to take part in the study.

### Data collection

Two different data collection methods were used in this study: FGDs for the adolescent girls and qualitative interviews for the adult key informants. FGDs are useful when developing an intervention model because they allow for synergism and spontaneity between participants, as well as facilitating the snowballing of ideas (26).

For both logistical and scientific reasons, we used individual qualitative interviews with the adult informants: it was more practical to meet with these busy people at their convenience on a one-to-one basis, and we were specifically interested in their own personal views and experiences, as opposed to the more normative attitudes often expressed by a group (27).

Our first task in developing the FGD and qualitative interview protocols was to review the literature (4, 9, 28, 29) in order to identify some of the potential facilitating factors and barriers associated with physical activity among adolescent girls in similar communities in South Africa. From this, we developed a draft set of questions for the girls and the adult informants, which we discussed and adapted with input from the three trained, highly experienced, local female fieldworkers (JM, SN, and NM) who would collect the data. Adaptations to the questions were mainly concerned with improving clarity, but we did include an additional question about peer pressure from other girls. The adapted questions were then translated into Shangaan (the local vernacular), piloted, and final, minor amendments were made. This process aimed to ensure that the questions we used were locally contextualised and appropriate for the setting. The topics covered during the discussions with the girls and the adult key informants are presented in Table 1. Note that the focus in this study was specifically on physical activity; we did not examine nutritional factors as an issue affecting body weight.

**Table 1:** Topics covered in FGDs and key informant interviews

Focus group discussion topics: adolescent girls
Perceived relationship between physical activity and health Reasons for participating or not participating in physical activity Satisfaction/happiness with their own level of physical activity Influence of school, peers, and gender on physical activity What sort(s) of physical activities they do and would *like* to do
Qualitative interview topics: adult key informants
Physical activities that are organised for girls and boys (together and separately) Sports competitions: who participates, who organises, how often, etc. Gender and age differences in expectations and practice of physical activity Challenges faced in organising physical activities, and how these are dealt with How best to increase girls’ participation in physical activities

Data were collected in June and July 2011. The qualitative interviews took approximately 45 min each, and the FGDs lasted between 60 and 90 min. They were all conducted in private rooms on school property. Healthy refreshments were offered to all informants after the interviews; no other incentives were provided. The qualitative interviews and FGDs were digitally recorded (with the consent of the participants), transcribed, and translated into English for analysis.

Confidentiality was guaranteed for the interview respondents, but since confidentiality cannot be guaranteed for FGD participants, they were requested to respect each other's privacy, and not to discuss in other settings anything brought up during the discussions.

### Data analysis

**Fig. 1:**
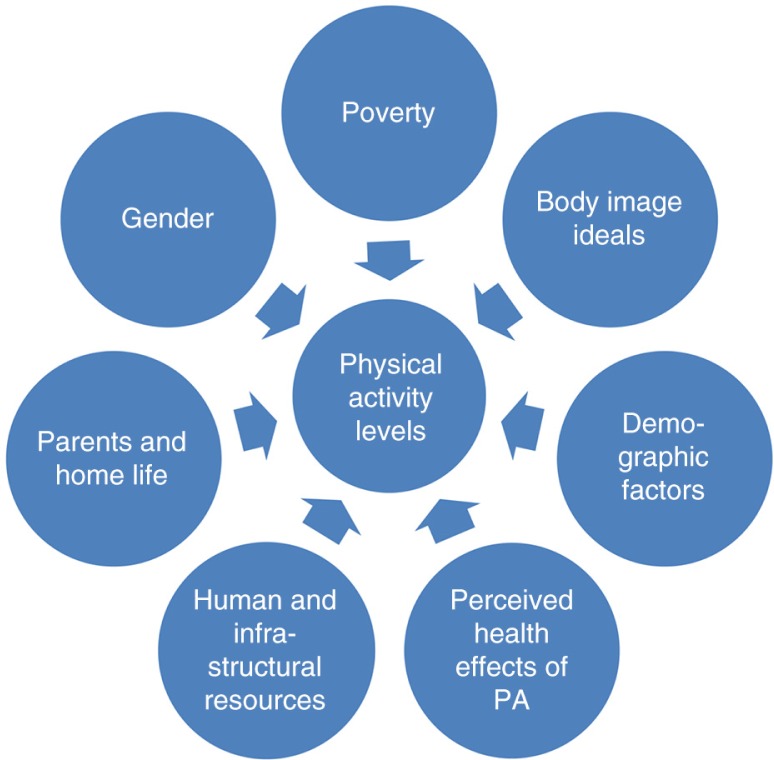
Seven themes associated with girls’ levels of physical activity.

Our analytical approach was based on social phenomenology, a ‘descriptive and interpretive theory of social action that explores subjective experience within the taken-for-granted, “common-sense” world of the daily life of individuals’ (30, p. 81). On this basis, we conducted a thematic analysis (31) using OpenCode 3.4 software (32). A series of steps was taken in the process. First, a code manual was developed, first for the FGDs and then for the qualitative interviews. These initially included a set of *a priori* codes based on our literature review (such as peer pressure, parental influence, and impact of poverty), but with additional codes subsequently included as they were inductively identified by JK during initial readings of the data (such as social status, boys, and influence of television). A set of broader themes was then developed from these codes, whereby codes that shared some key characteristic were brought together into a larger thematic group, for example, gender, human and infrastructural resources, and body image ideals. The seven themes that emerged constitute the framework that is presented in the Results section (see also Fig. 1). Some of the themes apply exclusively to what the girls said, some of them apply just to the adult key informants’ comments, and some of them are cross-cutting. In those cases where both groups talked about the same issue (such as the role of parents, and the limited available human and infrastructural resources), we were able to triangulate the data and develop an understanding of the issue from the two different perspectives. Finally, a narrative text was then built for each theme, supported by similar and contrasting views or experiences, and illustrated through relevant quotes.

### Ethics statement

Both the participating girls and their parents or legal guardians gave signed informed consent to participate. Ethical approval to conduct the study was granted by the Human Research Ethics Committee of the University of the Witwatersrand (Clearance Certificate M110211), by the Mpumalanga Department of Health Research and Ethics Committee, and by the Mpumalanga Department of Education.

## Results

Responses to two of the open-ended questions from the girls’ FGDs are included in Table 2. These questions were: 1) *Describe the sorts of physical activities that teenage girls here undertake* (we probed for formal, school-based activities; informal, leisure-time activities; and household chores); and 2) *Do you have any suggestions for activities, sports, or games that you think teenage girls in this area would like to take part in*? The top 10 responses are given for both these questions, ranked in order of preference, alongside the number of times they were mentioned. The most notable finding from these data is the domination of household and agricultural chores in the list of activities that the girls currently do (6 out of the 10 responses), whilst enjoyable group leisure-time activities, such as dance, netball, and ladies soccer, dominate the list of what they would *like* to do.

**Table 2:** Sports and other activities that girls undertake, and those they would like to do, ranked in order of preference

Sports or other activities that girls undertake (number of times mentioned)	Sports or other activities that girls would *like* to do (number of times mentioned)
Ladies football (9)	Dance (9)
Fetch water (9)	Netball (8)
Gardening (6)	Ladies football (5)
Cooking (6)	Tennis (4)
Cleaning (6)	Music (3)
Running/athletics (6)	Running/athletics (3)
Dance (5)	Volleyball (2)
Netball (5)	Javelin (2)
Fetch firewood (4)	Shot put (2)
Grind maize meal (3)	High jump (2)

The remaining data, from both the FGDs and the qualitative interviews, produced the seven thematic areas that were associated, either as facilitators, barriers, or both, with participation of the girls in physical activities. These themes are represented visually in Fig. 1.

Note that although the themes are presented here as discrete entities, several of them are overlapping. Poverty and gender, for example, play important cross-cutting roles, but they also stand as important themes in their own right.

### Poverty

Poverty is both a facilitator and a barrier for teenage girls to take part in physical activities. On the one hand, it provides an important incentive for the girls, most of whom lack access to cash, to participate in sports competitions. Much of their motivation to engage reportedly springs from the possibility of winning money and other prizes in these competitions – through sponsorships by corporate organisations, for example – where the winners (but no other participants) receive prizes.

‘C’ [large organisation] made a sponsored competition. They say the school that is going to obtain Position One, they are going to give them a jersey and ball and they also have a [financial] prize. [Qualitative interview, Female Sports Organiser, 35 years]

On the other hand, data from the FGDs also point to the social stigma associated with coming from poorer backgrounds in this community, which, we found, has complex and negative implications for engaging in sports and other physical activities. The following statements from one of the FGDs illustrate this stigma, as well as the pressure that poorer girls can face from their peers.

You find that we are girls and we are friends but if … she doesn't want to be our friend, we will tell the others about her background, that she's poor, and they don't have food at her home. [FGD 2, 16- to 19-year-olds]

Girl 1: They are friends. They wear jeans [but] one of them, she's not wearing jeans. When they go out, they will tell her that, ‘We are not going with a person who is wearing a skirt. If you want to go with us, just go and wear jeans’. But they know that this person has never ever worn jeans in her life. [They all laugh]

Girl 2: And that girl she will have a pressure. [FGD 2, 16- to 19-year-olds]

The process by which this pressure can act as a barrier to physical activity was explained by our informants through the following series of steps: girls living in poorer households are more likely to have to walk to school as opposed to getting there by mini-bus taxi (these provide a relatively cheap means of transport along pre-defined routes); and they are usually obliged to help out at home, either with housework or gardening.

At home we have a domestic worker. When I go home I don't have anything to do because the helper has done everything. When I come home I just bathe and go to bed. In the morning I wake up and go to school. But some when they [poorer girls] go home, they have to cook, clean the house and wash some dishes. [FGD 4, 16- to 19-year-olds]

As a result of their physically active lives, they may have well-developed muscles, and these may be seen by others as indicative of poverty. Girls with muscles can become targets for mockery and bullying.

Oh, they [the boys] laugh at them [the girls]. They told them that they have ‘mapotirisi’ [=muscles], and they have a hard skin because they work hard at home. [FGD 2, 16- to 19-year-olds]

This makes it a priority to avoid developing muscles, by, for example, remaining as sedentary as one's life situation permits. The wish is to emulate the more fortunate girls, economically speaking, who are reportedly proud of their un-toned bodies.

They [the girls whose families have domestic help, or a vehicle to drive them around] think they're better … and they think they are soft like an egg. [FGD 2, 16- to 19-year-olds]

Through this social process, poverty can act as a significant disincentive for girls to take part in sports and other activities.

### Body image ideals

The complexity of the social situation is further exemplified by the more or less mutually exclusive body image ideals towards which the girls aspire. While it is socially undesirable for a girl to be muscular, as discussed above, they do nonetheless face pressure to be thin; and to this end, some overweight girls are highly motivated to lose weight, even though they find it difficult.

They [overweight girls] do their best to find themselves as us [thinner girls]. They do some exercises, but they don't lose the weight. [FGD 2, 16- to 19-year-olds]

The motivation for overweight girls to work towards this thin ideal is based on a range of issues. Importantly, no mention was made by any of the girls about losing weight for health reasons. Rather, the motivation, in a number of cases, was to avoid unpleasant censure or humiliation, either from boys or from other girls.

If you are having big buttocks they [other girls] laugh at you [girls laugh]. [FGD 3, 13- to 15-year-olds]

Sometimes they [boys] compare us [overweight girls] with baboons. [FGD 1, 13- to 15-year-olds]

Some of the girls have reportedly tried to lose weight specifically to increase their chances of attracting a boyfriend.

A person who is obese, she doesn't have a choice when it comes to boyfriends. [FGD 6, 16- to 19-year-olds]

Boys like portable girls, I mean a slender girl, not a fat one. [FGD 2, 16- to 19-year-olds]

However, and in contrast to the views just presented, there also appears to be strong, simultaneous social and cultural pressure for girls to be overweight. As with the pressure to be thin, this demand to be overweight came from boys as well as from girls, but either way, it represents a clear disincentive to engage in physical activities.

When boys look at them [overweight girls] they feel crazy because they are fresh, but when they look at a person who is working or doing exercise they see an old girl while she's young. [FGD 2, 16- to 19-year-olds]

Many girls also ascribe to an ideal that appreciates ‘curves’ in a female body – that is, some extra fat. Further to this, there was the suggestion that girls who do not conform to this ideal may be teased or bullied on the basis that their ‘thinness’ indicates HIV infection.

Sometimes you find that the other girl is having some curves and you don't have it. If she saw you she is going to call you by all names saying that you don't have curves, you're thin as if you're HIV-positive, you don't have buttocks. [FGD 3, 13- to 15-year-olds]

### Gender

With respect to formal sporting activities, gender plays an important role in defining whether, how, and the extent to which girls are, or are not, physically active. While sports facilities are generally quite poor in the area, facilities tend to be better for boys than they are for girls.

Our soccer pitch for boys is good, but for girls it's not good because the [netball] ground is inside the school yard and their place is too small. [Qualitative interview, Male Sports Teacher, 44 years]

Furthermore, sports competitions for boys are better organised, and better funded.

Mr ‘M’ is an owner of S [business] and he is sponsoring all the schools. He concentrates on the under-21s only, and he is doing it for boys only. Girls are suffering when it comes to competition and they don't have enough games, their games are very scarce …. To have sponsor for girls it is scarce, especially for netball or ladies soccer. Girls are left behind but for boys everything is going well. [Qualitative interview, Male Sports Teacher, 44 years]

In these ways, gender acts as a barrier for the girls to gain access to facilities and competitions in which they may be physically active.

### Parents and home life

Parents of some girls actively support participation of their daughters in team sports, for example, by providing money to travel to competitions. However, the overriding sense from the respondents, and in particular the adult key informants, was of parents wanting to keep their girls near to or at home, where they are safe and where they can be properly supervised. Consequently, many girls are held back from engaging in physical activities. The root of parental thinking here is at least partially a desire for their children to complete their education and thereby escape from poverty.

One other thing that makes the parents to be strict, you find that a parent grew up in a poverty family and she doesn't want her kids to grow up like her, because she knows the poverty, she wants them to be educated. They don't want their kids to suffer like they have, and that is why they are strict. [FGD 6, 16- to 19-year-olds]

The adult informants explained that girls in the area are vulnerable to sexual violence, but some also actively, if secretly, seek out sexual relations. Any sort of sexual activity, whether coerced or not, can be very risky for their education, as girls who fall pregnant are usually obliged to leave school.

There are people who are observing when we go to work or other places; when it is a woman and she is left alone, they are thinking of going there to rape her. [Qualitative interview, Male Youth Leader, 28 years]

They are sexually active … she prefers to go for a date and say I am going for an exercise, meanwhile she is on her date. [Qualitative interview, Male Youth Leader, 33 years]

As a result of all these concerns, parents often make their girls stay at home after school or on weekends, where they cannot meet boys or be hurt, but where they are also unable to socialise or have access to any sort of organised physical activity, and where they are often sedentary.

When they arrive and find that their parents have cooked and the food is ready, they will eat and they turn on the television. Nowadays they are having DSTV [cable TV]; they will stay and watch the Nigerian stories. And when you are watching those stories, you will not move, you can stay the whole day watching those stories. [Qualitative interview, Male Sports Teacher, 44 years]

### Demographic factors

Two major demographic factors were identified as relevant to physical activity: age and nationality. With regards to age, physical activity was widely seen by the respondents as something for younger girls more than for the older ones.

Even at school when you want to participate in athletics, they will tell you that you are over-age, so you become discouraged. [FGD 4, 16- to 19-year-olds]

Others are saying that they are not exercising because they are old. [FGD 1, 13- to 15-year-olds]

The adult key informants also spoke of older teenagers starting to spend increasing amounts of time and energy seeking out alternative pastimes, such as alcohol, marijuana, and/or sex, rather than more healthy physical activities.

With respect to nationality, formal identification documents are required as proof of age for participants in youth sports competitions in order to ensure that they play within the correct age group. This requirement can be challenging for any teenager whose parents do not have proper documentation for them, a problem which is especially evident for those whose parents are unregistered immigrants from Mozambique. Many of these teenagers have no legal documentation, so would be unable to prove their age for a sports competition. This in turn has implications for their motivation and participation, as well as creating logistical difficulties for both organisers and participants.

You have a team that you have trained and it's good, and you are expecting that team to go and play, and then you find that they don't have documents … so we need to jump up and down going to Home Affairs in order to register them to have documents. [Qualitative interview, Male Sports Teacher, 44 years]

### Perceived health effects of physical activity

A potentially important influence on whether or not someone is physically active is the perceived impact of that activity on their health. For example, if someone perceives physical activity as likely to be beneficial to his or her health, he or she may be more inclined to become or stay active. Some of the girls in our FGDs described such potential health benefits of physical activity, as well as the risks that could be associated with inactivity.

People who are not working hard, that makes them have high blood pressure at the end. [FGD 4, 16- to 19-year-olds]

When she is physically active it will make her that even when she grows older she will be continuing to do that exercise and she will not get older very soon. It will take time for her to grow older; she will always look good in her body. [FGD 5, 13- to 15-year-olds]

However, other girls suggested that they thought there were health risks associated with physical activity. These perceptions could act as a disincentive to engage in physical activities.

It [physical activity] might cause her high blood pressure. [FGD 2, 16- to 19-year-olds]

### Human and infrastructural resources

The adult key informants currently working in sports and other youth activities are individually very well motivated and committed, but they receive negligible financial support for their work.

The amount [of money] that you had been allocated for the sport doesn't fit your budget, then we end up not doing our job in a good way. [Qualitative interview, Male Sports Teacher, 37 years]

In our [volunteer-run] organization none of us are working [i.e. in paid employment]. It is very hard to save money and be able to use it …. Even when we take the adolescents out, they must eat something. [Qualitative interview, Male Youth Leader, 33 years]

Further, some of them reportedly feel isolated from each other and from their colleagues in their sports-related work.

I am alone in this community who is focusing in this youth organization. If you can be there assisting me, […] it will be very helpful. [Qualitative interview, Male Youth Leader, 28 years]

The other educators are here just to work their job, but when it comes to sport, they have nothing to do with it. [Qualitative interview, Male Sports Teacher, 44 years]

In some cases, it also emerged that sport or other non-classroom activities are not prioritised by the school authorities. This applies not only to the provision of equipment and facilities but also to allocation of time for sporting and other physical activities.

In soccer I am surprised when I realize that at schools they don't have soccer balls until our youth organization gets involved. They have soccer on their schedule but I don't see them playing. [Qualitative interview, Male Youth Leader, 33 years]

In general, sports nowadays is far behind, and the thing that makes learners not to be active – not that they don't want to participate – is the school … because they don't give them a chance to do exercises, they always want them to be in class and if you are on the sports committee then is like there is a type of war between you and the management. [Qualitative interview, Male Sports Teacher, 37 years]

Sports and other playing facilities for girls are generally poor or absent in the Agincourt area. Sports teachers spoke of the dangers of snakes and broken bottles on the playing areas, and of location problems as serious inhibitors.

When they [girls] are busy playing [netball], you find that a car of a teacher wants to go out and it disturbs them. Sometimes when they are busy playing, you find that some of the students are coming out of the class and they will pass via the ground where they are playing, and they get disturbed. [Qualitative interview, Male Sports Teacher, 44 years]

## Discussion

This study of factors that facilitate and inhibit physical activity among rural South African adolescent girls has identified seven themes, as presented in the text above and in Fig. 1. Links can be seen between several of these themes: for example, poverty and gender issues combine to create conditions whereby concerned parents seek to restrict their daughters’ out-of-school activities, and thereby their ability to engage in physical activities. Poverty also affects body image, and subsequently girls’ willingness to be physically active. Other themes influence girls’ physical activity levels more directly, the lack of sports infrastructure being perhaps the clearest example. The study has also shown that there is an association between normative values, body image, and physical activity, through the identification of clear, culturally embedded disincentives for being active.

These different factors reflect the three major, overriding contextual issues – gender inequality, rapid social transitions, and chronic poverty (23) – that were identified at the start of this paper. These issues combine to produce a challenging environment in which to attempt any sort of intervention. Health promotion interventions within this context need to acknowledge and take these overriding issues into account: recognising them is a core part of ensuring that any intervention we may design can be seen as ‘culturally competent’ (22). Although this may be challenging, developing and implementing culturally competent physical activity interventions in low- and middle-income settings has been shown to be feasible. The Agita São Paolo Programme in Brazil (33), for example, has demonstrated the potential of an intervention which explicitly sought to adapt to local cultural norms.

Furthermore, as indicated above, while contextual issues are defined by powerful social and economic structures, they are also dynamic and may therefore be somewhat malleable. Efforts to address both the contextual issues and the factors such as the seven themes described above should be seen as part of a broader process of community development and empowerment. Within this analytical framework, it is also important to recognise and work with the concept of social identity during the development of interventions to promote physical activity. Individuals are often simultaneously members of different peer groups, each of which comprises a specific part of a person's identity, and each of which can be an important factor in shaping their behaviour. Thus, efforts should be made to create peer group norms that support physical activity and promote physical activity within the context of their intergroup behaviour. This premise has contributed to the development of own model to promote physical activity.

Table 3 comprises the seven themes identified above and summarises the effects of issues inherent in each theme on physical activity. We also indicate which of these issues may be modifiable – most of them are modifiable, at least to some extent – and, within the context of the data presented above, we then propose possible interventions that could ameliorate each of the modifiable barriers to physical activity in this setting.

A number of studies on physical activity point to the importance of understanding the needs and concerns of individuals, as well as the roles of social and practical support from families, peers, schools, and communities (34–38). Based on this literature, we have synthesised the possible interventions identified in Table 3 into a model for promoting physical activity among these adolescent girls based on *supply-side* and *demand-side* principles. The supply side can be characterised as relating to individuals, schools, or organisations that provide the opportunities for physical activities, while the demand side represents the girls and their parents, who play a major role in determining whether or not their daughters take up these activities.

**Table 3:** Possible interventions that could ameliorate the impact of the barriers to physical activities associated with the seven themes

Theme	Effect on PA	Modifiable?	Possible intervention
1. Poverty	Stigma of poverty leads to inactivity: muscles are a sign of poverty, as poor girls have to work at home. Muscles are therefore to be avoided by being sedentary.	Not as part of a PA intervention but can be used as a lever to promote PA	Provide incentives for participation in sports, on a conditional cash transfer principle.
2. Body image ideals	Conflicting peer group perspectives of both benefits *and* disadvantages of being thin and overweight.	Yes	Develop empowering messages about healthy weight and body image.
3. Gender	Fewer opportunities for girls to be physically active. Girls’ sports facilities are invariably worse than that for boys.	Yes Yes	Promote girls-only activities, for ALL girls. Promote female sports teachers and youth leaders. Increase resources for acquiring and maintaining equipment and facilities (see also 7 below).
4. Parents and home life	Parental fears of sexual activity of their daughters (coerced or not) and pregnancy; they want to keep them at home, where they are safe, and often physically inactive.	Yes	Encourage parental engagement and attendance at sports events where their daughters are participating.
5. Demographic factors	Older teenagers tend to exercise less The need for national identification documents can make it hard for undocumented migrants to participate in organised sports.	Yes No	Offer the possibility for physical activities that older teenagers will *want* to engage in.
6. Perceived health effects of PA	Perceived benefits of PA could lead to more activity; perceived risks of PA could lead to less activity.	Yes	Design appropriate health messages about PA.
7. Human and infrastructural resources	Poor infrastructure and feelings of isolation reduce possibilities for the provision of organised physical activity.	Yes	Provide training and support for youth leaders and sports teachers. Increase resources for acquiring and maintaining equipment and facilities.

PA=physical activity.

**Fig. 2:**
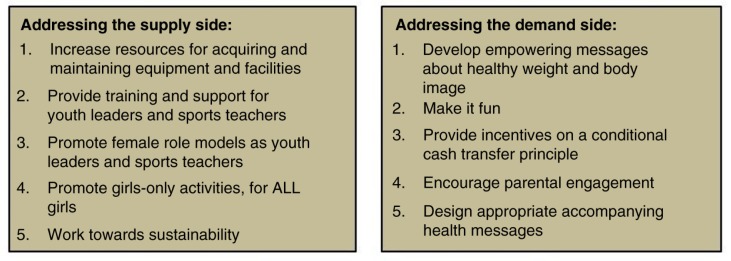
A model for promoting leisure-time physical activity among rural South African adolescent girls.

The model presented in Fig. 2 provides a series of practical, actionable steps that can be taken, within this supply-side and demand-side framework, to promote leisure-time physical activity for adolescent girls. While obstacles and challenges are to be expected along the way, we believe that, collectively, the issues we have identified constitute a potentially effective toolbox for physical activity intervention development in a rural community.

Our study community is in the midst of a process of rapid social, demographic, and epidemiological transition, with chronic poverty and gender inequalities reflected in many aspects of people's daily lives (23). Since these broad contextual factors are common to many other parts of sub-Saharan Africa, we believe that the model we present for promoting physical activity within rural South Africa could also be applied in other, similarly resource-constrained settings, both within South Africa as well as elsewhere on the continent.

### Supply side

Five broad issues needing attention on the supply side are identified from this study. These focus on the needs of the individuals, schools, and organisations that provide and facilitate the activities.

#### Increase resources

The sports teachers and youth leaders we interviewed faced very tight resource constraints, and they had correspondingly poor facilities within which to work. This is by no means unique to the Agincourt sub-district, with other rural and urban parts of South Africa experiencing similar challenges (28, 29). Several of our adult respondents dreamed of having a range of facilities at their disposal, such as tennis courts, a hockey or cricket pitch, or even a full-fledged sports centre, which could house diverse activities. The increased prominence recently given to physical education within the new National CAPS (Curriculum and Assessment Policy Statement) curriculum suggests that additional resources may be made available for schools, but these would not provide such a high grade of facilities. One way or the other, however, improvements to facilities and equipment are urgently needed, particularly for activities undertaken by girls. Furthermore, any improved facilities and equipment need to be maintained, protected, and managed by members of the community, thereby facilitating long-term sustainability.

#### Provide training and support for youth leaders and sports teachers

Our adult key informants reported a strong sense of isolation in their work, alongside a general lack of support for what they were trying to do. There is a clear need to provide this support, as well as adequate training for youth leaders and sports teachers (39). Moreover, it would be helpful to establish networks of individuals who are working in given geographical areas, so as to provide ongoing peer support (40). Furthermore, we found indications that some school administrators regard sports and other physical activities as a distraction from pure academic work. The value of physical activity in schools should therefore be emphasised, both for physical health and for promoting pupils’ attention and learning capacity in class (41, 42).

#### Promote female role models in sports

Although our sampling framework was purposive rather than systematically representative, it was nonetheless striking that only one of the seven key informants was a woman. Anecdotal evidence suggests that this proportion is broadly reflective of the gender (im)balance among sports teachers and youth leaders in the area. Given this scenario, it would be important to actively promote women into coaching and role model positions. The 2007 Healthy Active Kids South Africa report card has identified a general lack of school ‘champions’ or coaches to promote sports participation in the country (43), and it would be particularly important for girls who do not have suitable female role models to have such women made visible and accessible to them.

#### Promote girls-only activities, for ALL girls

We found that most of the group activities and competitions specifically targeted boys, with very few opportunities for girls. Given the established gender division in sports activities that we observed – with boys classically playing football and, where viable, girls playing netball – promoting girls-only activities and competitions is of vital importance. These activities should particularly target girls who are currently too sedentary but could become active given the right conditions, which would constitute an important public health gain. Girls-only activities should therefore be for ALL girls, with care taken to provide activities that would interest girls of all ages and abilities.

There is a global movement to empower girls and women through sport, with organisations such as ‘Women Win’ promoting work in Africa, South America, and Asia that aims to improve the health and well-being of girls, as well as their social and economic status, through sporting and other physical activities (40). Some of the lessons learned from these efforts include the importance of designing sports programmes that really meet the girls’ needs, ensuring that the coaches are well trained, and making certain that the girls feel safe in the programme (40). These points should be fully incorporated into girls-only activities.

#### Work towards sustainability

In order for any physical activity intervention to be sustainable, the primary responsibility for its maintenance and management must come from the community (39). This requires some degree of leadership, motivation, and mobilisation: the Community Health Intervention Programmes in the Western Cape, for example, found that increased community ownership of, and responsibility for, the programme was critical to its sustainability (39).

A first step towards sustainability would be, through a process of community mobilisation, to identify local people willing to take on this responsibility (39). The dedication of our adult key informants suggests that such people are indeed available, but external resources are needed to facilitate their work, and in the absence of any substantial state support, we propose that these could be brought in through non-governmental organisations working in the area and/or through sponsorship from local businesses. With the latter, business people could be encouraged, for example, to branch out into supporting girls-only activities and competitions, thereby complementing the boys-only competitions that are currently supported. It would be important to make such ‘corporate social responsibility’ activities attractive for sponsors, so that they feel they have something to gain by supporting them, whether socially or economically. Successful sponsorship could provide a win–win situation, whereby the programme is effectively supported, while the sponsoring businesses also benefit from good public relations through their contribution to the community.

### Demand side

We identified five key areas that require attention on the demand side. These are concerned with the girls themselves and their parents, and they should be addressed in order to make any intervention appealing to the target group.

#### Develop empowering messages about healthy weight and body image

Adolescent girls are faced with mutually exclusive ideals of what is regarded as attractive and unattractive by their female peers, by the boys they desire, and by their culture. If they are active or thin, for example, they may be taunted for being poor or for having HIV, whereas if they are overweight, they could be compared with baboons, or laughed at for their large buttocks.

Such contradictory perspectives have been reported in previous studies about body image among South African women. Puoane et al. (44) found that ‘in a black culture … a woman is admired if she has some padding over the hips, [while] women who are exposed to media images, which portray thin women as attractive, become confused’ (p. 14). Consequently, ‘they tend to want to be both. On the one hand they want to be what they are supposed to be according to their own cultural values, on the other hand they want to conform to values of other cultures’.

Another study, conducted in the Western Cape, found that 69% of 513 adult female informants associated a thin figure with a person infected with HIV, or suffering from AIDS, while only 10% thought that a thin figure symbolised health (45). These women said they would prefer to be overweight and at risk of acquiring cardiovascular diseases, rather than being thin and stigmatised as a person infected with HIV or having AIDS. Such attitudes towards a larger female body have been described by Puoane et al. as ‘symptomatic of socioeconomically disadvantaged communities in which thinness is tantamount to poor health and misery’ (46, p. 33).

This all highlights the considerable difficulty of effectively bringing about a healthy change in attitudes towards being overweight in settings where poverty and HIV are pervasive. Some of the girls we interviewed wanted to become physically active, or to lose weight, but we noted that their motivation was more negative (to avoid humiliation) than positive (to achieve any sort of health benefit, or to feel good). This conflict should be borne in mind when seeking to develop empowering messages that encourage girls to try to keep a healthy weight and to be physically active.

#### Make it fun

A significant part of the motivation for adolescent girls to participate in physical activities is to ‘make new friends [and] have fun’ (47). It is important that activities are marketed for these reasons. Table 2 shows that many girls are engaged in various household chores, some of which are quite physically demanding. Although the physical activity inherent in these activities is likely to have important health benefits – something the girls should certainly be made aware of – they do not appear on the side of the table representing activities that girls would *like* to do. This latter list is dominated by far more enjoyable activities, such as dance, netball, and ladies soccer.

While many teenage girls may find such activities enjoyable, there are others who simply do not want to engage in activities that make them sweat, and it may be counterproductive to insist that they do so. Lighter intensity activities are needed for such girls, such as music and dance (as in Table 2). These activities would involve some movement, thereby bringing about health benefits, while simultaneously offering potentially valuable social and life skills development.

#### Provide incentives on a Conditional Cash Transfer principle

Much of the girls’ discourse in the FGDs focused on the need to beat poverty as a first step towards being happy, on the premise that you cannot be happy if you are poor. This appeared to provide an incentive for some of them to participate in competitions, where a small financial reward was given to the winners. But encouraging sporting participation on this premise will only benefit the most athletically talented girls, who are intrinsically less likely to be overweight or obese.

In contrast, facilitating activities for the less athletic majority could reap significant population-level benefits (47). One way to increase physical activity levels among the less active girls could be a conditional cash transfer (CCT) approach. CCT schemes consist of welfare payments made to families or individuals who fulfil certain requirements, such as enrolling in school or vaccinating their children. A recent study in Malawi, for example, found that a CCT can reduce HIV infections in adolescent schoolgirls by paying them to stay in school (48). The largest CCT in the world is the Bolsa Familia Programme in Brazil, which provides financial aid to poor families on the condition that their children attend school and are vaccinated. The programme has reached some 46 million people and has contributed significantly to reductions in inequality and extreme poverty in the country (49). While recognising the administrative and logistical challenges of this approach, we believe that the CCT principle could be extended to the promotion of physical activity, with small payments made, or vouchers for healthy foods provided, to girls who participate in activities or competitions, regardless of their performance.

#### Encourage parental engagement

Parental engagement has been recognised as a key component in the successful promotion of physical activity for youngsters in European countries (50, 51) as well as in Australia (52). The same clearly applies in South Africa, where efforts must be made to ensure that parents understand the health benefits of physical activity if they are to believe that participation would be in their daughters’ best interests. However, and perhaps to a greater extent than in these other settings, it is essential to take into account parents’ legitimate concerns about the well-being and safety of their daughters. Our informants spoke of the dangers of rape in the area, which is a very significant problem in much of South Africa (53). All efforts must be made to ensure the safety of girls who take part in these activities. One approach could be to encourage parents themselves to attend, which would show support for their daughters’ participation while simultaneously reducing the girls’ risk of coming into situations where they might be attacked, or where they could willingly engage in sexual activity.

#### Design appropriate accompanying health messages

The nationwide Youth Risk Behaviour Survey of 2008 found that a substantial group of school pupils do not take part in physical activities because they are unaware of the health benefits (10). Our findings concur with this, with a number of misconceptions identified about the negative impact of physical activity, such as high blood pressure or pain when one stops. Addressing such misconceptions must be the central component of any social marketing efforts to promote a physical activity intervention.

### Study strengths and limitations

The strength of the findings from this study is enhanced by the unique mix of professional and personal backgrounds in the study team, which provide a rich combination of ‘insider’ and ‘outsider’ perspectives, and which we believe give us cultural competence (22) for this setting. We include members who have lived in the Agincourt community for their whole lives, scientists who have been studying the health and demographics of people in the area for over 20 years, and scientists with expertise in physical activity and qualitative research methods. Methodologically, we used two different qualitative research techniques to investigate the issue, and these came up with broadly complementary findings. Taking Guba's four criteria for trustworthiness in qualitative research (54), we believe that our findings are 1) credible, insofar as the findings have been unreservedly accepted by all those in the study team, including those with longstanding experience of the Agincourt area; 2) transferable, because, although our findings are intimately tied to the time and the context from which they were derived, the core characteristics of Agincourt are shared by many other contexts, both in South Africa and elsewhere on the continent; 3) dependable, as we report full details of the processes within the study, thereby enabling a future research team to repeat the work in a similar way; and 4) confirmable, since we – a multi-disciplinary team of researchers, closely connected to the community we were researching – were able to triangulate the findings between two groups of informants.

It is also important to note that while the number of FGDs was decided before data collection commenced, and the number of qualitative interviews was defined by the availability of suitable informants, we did reach saturation with regards to the major emerging themes.

Three possible limitations do need to be acknowledged. First, the study was conducted in one specific site in rural South Africa, so questions could be raised about its applicability in other settings. However, our findings are broadly consistent with many of the issues identified in other studies, both from South Africa and internationally. These include issues on both the supply side and the demand side, ranging from the dedication of sports teachers and youth leaders and the need for them to receive training and support, to the challenges faced by adolescent girls in relation to body image ideals, to the importance of parental support in getting girls to be physically active. The model we present could, therefore, be transferable to other similar settings in South Africa as well as to other sub-Saharan African and resource-poor countries.

Second, we have no way of knowing whether any bias may have been introduced into the FGDs through self-selection. It is possible, for example, that girls from wealthier backgrounds or those who were more physically active could have been over-represented. If there was any such bias in the dataset, however, triangulation between what the girls said with what the adult key informants said indicated no major contradictions between the two groups. This suggests that any possible bias was not sufficient to fundamentally affect our analysis or interpretation of the data.

Third, our sampling framework was purposive rather than systematically representative, and through this approach we recruited only one female adult key informant for the seven qualitative interviews. We may therefore have obtained data with a male bias. Anecdotal evidence suggests that these proportions were broadly characteristic of the gender (im)balance in the world of sports teachers and youth leaders in the area, and as such, any bias that is found in the data probably reflects the overall demographic make-up of adults working in this area. However, the significant under-representation of female sports teachers and youth leaders in our data necessitates that extra efforts be made in any future evaluation of the model to ensure that the views and experiences of such women are systematically taken into account.

## Conclusions

There is an urgent need to develop effective physical activity interventions in South Africa as one of several means to counter the rapidly expanding epidemic of NCDs in the country. A key demographic group for this is adolescent girls, whose collective health profile places them at a disproportionately high risk.

Through the use of a supply-side and demand-side framework, we have provided a model with clear action points aimed at assisting in the development of what we believe would be an appealing and effective physical activity intervention for adolescent girls living in resource-constrained environments. The broad congruence of our findings with those from a range of studies in other settings suggests that the model may be applicable elsewhere in South Africa, as well as in other African and resource-poor countries that are undergoing health and social transitions alongside an upsurge in NCDs.
